# Explaining Orientation Adaptation in V1 by Updating the State of a Spatial Model

**DOI:** 10.3389/fncom.2021.759254

**Published:** 2022-02-18

**Authors:** Shaobing Gao, Xiao Liu

**Affiliations:** ^1^College of Computer Science, Sichuan University, Chengdu, China; ^2^Tomorrow Advancing Life Education Group (TAL), Beijing, China

**Keywords:** neural adaptation, orientation tuning curve, receptive field, image statistics, V1

## Abstract

In this work, we extend an influential statistical model based on the spatial classical receptive field (CRF) and non-classical receptive field (nCRF) interactions (Coen-Cagli et al., 2012) to explain the typical orientation adaptation effects observed in V1. If we assume that the temporal adaptation modifies the “state” of the model, the spatial statistical model can explain all of the orientation adaptation effects in the context of neuronal output using small and large grating observed in neurophysiological experiments in V1. The “state” of the model represents the internal parameters such as the prior and the covariance trained on a mixed dataset that totally determine the response of the model. These two parameters, respectively, reflect the probability of the orientation component and the connectivity among neurons between CRF and nCRF. Specifically, we have two key findings: First, neural adapted results using a small grating that just covers the CRF can be predicted by the change of the prior of our model. Second, the change of the prior can also predict most of the observed results using a large grating that covers both CRF and nCRF of a neuron. However, the prediction of the novel attractive adaptation using large grating covering both CRF and nCRF also necessitates the involvement of a connectivity change of the center-surround RFs. In addition, our paper contributes a new prior-based winner-take-all (WTA) working mechanism derived from the statistical-based model to explain why and how all of these orientation adaptation effects can be predicted by relying on this spatial model without modifying its structure, a novel application of the spatial model. The research results show that adaptation may link time and space by changing the “state” of the neural system according to a specific adaptor. Furthermore, different forms of stimulus used for adaptation can cause various adaptation effects, such as an a priori shift or a connectivity change, depending on the specific stimulus size.

## 1. Introduction

Adaptation is the process by which neurons in the brain's sensory pathways adapt signals to the changing world (Carandini, 2000; Carandini et al., 2005; Manookin and Demb, 2006; Clifford et al., 2007; Kohn, 2007; Teich and Qian, 2010; Webster, 2011; Solomon and Kohn, 2014; Snow et al., 2016; Quiroga et al., 2019). Experiments show adaptation effects in most sensory systems at multiple levels, from neuronal processing to perception (Kohn, 2007; Maravall et al., 2007; Solomon and Kohn, 2014). Adaptation is essential because it allows sensory neurons encoding the world more efficiently and enables us to perceive the surrounding environment across a more extensive range (Solomon and Kohn, 2014; Snow et al., 2017; Weber and Fairhall, 2019; Weber et al., 2019). In this work, we focus only on neural orientation adaptation effects occurring in the primary visual cortex.

Adaptation to various stimuli normally results in very complicated neural responses in the visual cortex (Kohn, 2007; Wissig and Kohn, 2012; Patterson et al., 2013; Solomon and Kohn, 2014; Aschner et al., 2018; Coen-Cagli and Solomon, 2019; Yiltiz et al., 2020). The typical adaptation effects constrained to the CRF provoke suppression in individual neurons during stimulation, such as reducing neurons' sensitivity to all subsequent stimuli and ultimately leading to fatigue (Hammond et al., 1988; Giaschi et al., 1993; Carandini and Ferster, 1997; Dragoi et al., 2000).

In addition to suppression, adapting stimuli covering CRF can push away the optimal orientation of a neuron (Dragoi et al., 2002; Felsen et al., 2002; Wissig and Kohn, 2012; Patterson et al., 2013). Concretely, when a V1 neuron is adapted to the grating covering CRF with orientation 30–45 degrees away from its optimal orientation, adaptation would cause the adjustment of the neuron to deviate from the adapter (Dragoi et al., 2002; Felsen et al., 2002; Patterson et al., 2013). Similarly, when a V1 neuron is adapted to a grating covering its CRF at its optimal orientation, adaptation can reduce the overall orientation tuning curve (OTC) response of the neuron, and the maximum response reduction occurs at the neuron's optimal orientation (Müller et al., 1999; Dragoi et al., 2000; Felsen et al., 2002; Wissig and Kohn, 2012). Furthermore, in the situation that the orientation of an adapter covering CRF is orthogonal to the optimal orientation of the neuron, adaptation results in a typical enhancement phenomena (Wissig and Kohn, 2012; Solomon and Kohn, 2014).

However, the application of large-scale grating adapters covering both CRF and nCRF changes the OTC of a neuron in a manner that is quite different from the results of only stimulating the CRF (Webb et al., 2005; Tailby et al., 2008; Ghisovan et al., 2009; Wissig and Kohn, 2012; Patterson et al., 2013). For example, flank adaptation of the OTC of a V1 cell in which an adapter is stimulating both CRF and nCRF together will result in attractive shift in preference (Kohn, 2007; Wissig and Kohn, 2012; Patterson et al., 2013; Solomon and Kohn, 2014). A further difference between adaptation elicited by only stimulating CRF and adaptation elicited by simultaneously simulating both CRF and nCRF is that responses to the adapter orientation matched to the optimal orientation are suppressed when using the small grating stimulus, whereas responses are maintained or unchanged when using the large grating stimuli. However, adaptation in the orthogonal direction, whether only covering CRF or covering both CRF and nCRF together, always results in continuous or enhanced responses. Notably, these new adaptation results observed in V1 incorporating both CRF and nCRF have been previously observed in MT (Petersen et al., 1985; Priebe et al., 2002; Van Wezel and Britten, 2002; Kohn and Movshon, 2003, 2004; Krekelberg et al., 2006; Patterson et al., 2014). One recent study by Aschner et al. (2018) further showed that adaptation increases normalization signals when adapting stimuli consisting of orthogonal gratings are presented synchronously. Conversely, adaptation decreases normalization signals when adapting stimuli are presented asynchronously. Coen-Cagli and Solomon (2019) suggested a new functional role of normalization signals induced by nCRF that have a stabilizing effect on neuronal response variability (i.e., a type of adaptation of neuronal response). Yiltiz et al. (2020) indicated that adaptation can strengthen mutual suppression between subpopulations in the nCRF excited by the 2nd-order statistics of stimuli.

In summary, neurons in V1 adapt to recent stimulation experience according to different stimulus forms. These effects involve repulsing OTC away from the adapter, attracting OTC to the adapter, enhancing response, suppressing response, and retaining response (Wissig and Kohn, 2012; Patterson et al., 2013). These disparate observations pose major obstacles for mechanistic theories of how these results occur (Solomon and Kohn, 2014), and there is a present lack of a general framework for interpreting them (Kohn, 2007). Interpreting how neurons adapt may help us understand how our visual system processes temporal experience and how it interacts with spatial processing. This, in turn, may inspire novel computational algorithms (Medathati et al., 2016) that can process the dynamic information in a real-world. Furthermore, interpreting how neurons adapt also may help us infer the underlying cortical sensitivity of fMRI signals observed in both healthy people and patients (Lee et al., 2019).

In this work, we investigate all of the OTC adaptation results mentioned above by using a novel model that learns to predict the center-surround receptive field reactions through studying the natural image statistics (Cagli et al., 2009; Coen-Cagli et al., 2012; Snow, 2016; Snow et al., 2016, 2017). Our goal is to discover if this mathematical model may easily describe the diversity and stimulus specificity (for example, in the case of stimuli covering only CRF or covering both CRF and nCRF) of the OTC adaptation effects, and to understand how temporal adaptation alters the interactions between CRF and nCRF to produce these diverse results. To our best knowledge, the mathematical model was able to replicate all the OTC adaptation results found in neurophysiological studies (Wissig and Kohn, 2012; Patterson et al., 2013; Solomon and Kohn, 2014), especially the important adaptation effects using large grating stimuli covering both CRF and nCRF in V1.

In line with previous findings (Snow, 2016; Snow et al., 2016), our work further clarifies how the model presents diverse adapted responses under various visual stimulus sizes. In addition, we are contributing a new prior-based WTA working mechanism to explain why and how all of these OTC adaptation effects can be predicted by relying on this model. Furthermore, the existing information gained from raw images may be changed by presenting the model to a novel visual stimulation constituted of realistic pictures and physiologically used grating images. Our main finding is that the prior update in the model can clarify most of the recent findings on OTC in V1 after adaptation. However, we have further discovered that the observed attractive effects of adaptation using stimuli of large scale in V1 are implemented by the modification of the connection of CRF and nCRF. Specifically, the enhanced responses within the CRF resulted in non-specific suppression and the weakened surround suppression from the adapted orientation within nCRF getting together is the main factor necessary to understand the very novel attractive adaptation effect (Wissig and Kohn, 2012; Patterson et al., 2013; Solomon and Kohn, 2014).

## 2. Materials and Methods

**Figure 1:**
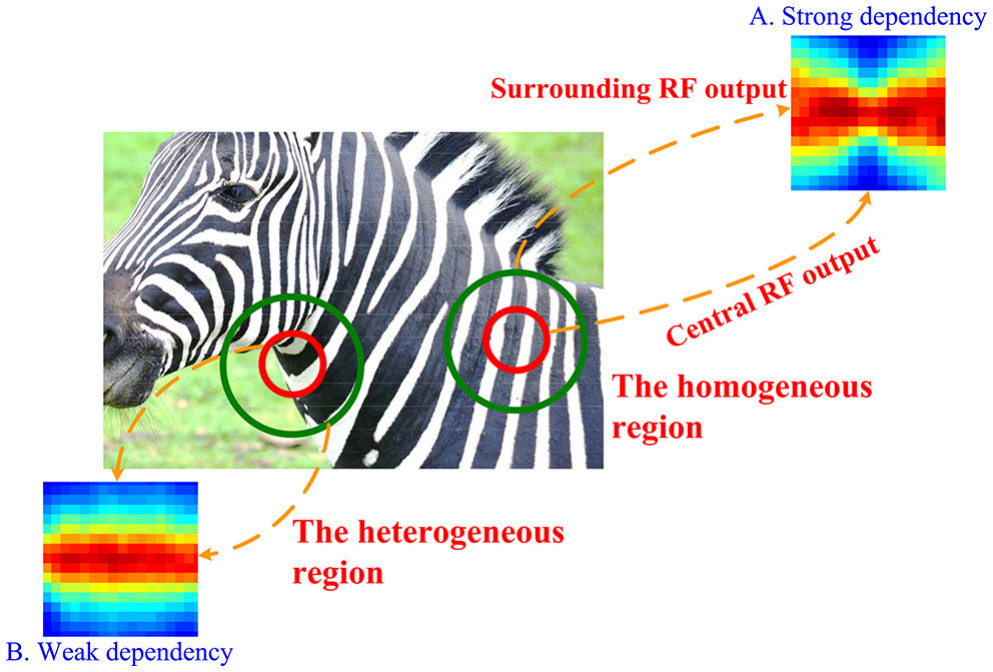
The CRF and nCRF outputs for a natural scene usually constitute nonlinear spatial dependency and independency. For a homogeneously textured region, the CRF and nCRF of a modeled V1 neuron, which are illustrated by red and green circles, respectively, receive similar features. Thus, two RF outputs produce the strong nonlinear dependency seen in **(A)**. In contrast, for a heterogeneous region, the CRF and nCRF of a modeled V1 neuron receive quite different features (i.e., the zebra stripes in the foreground and the grassland in the background), and thus the dependency between two RF outputs is quite weak, or the two outputs are statistically independent as in **(B)**. Adapted from Coen-Cagli et al. (2012).

The general idea of Mixture of Gaussian Scale Mixture (MGSM) model is shown in Figure 1 (Cagli et al., 2009; Coen-Cagli et al., 2012; Snow et al., 2016). For a homogeneous patch, there is a clear nonlinear spatial dependence on the outputs of two RFs (we refer to these simply as CRF and nCRF) shown in Figure 1A. The steerable pyramid filters are adopted as the V1-like RFs. For example, the CRF consists of a V1-like filter outputs with four orientations and two phases $C=c_{\theta }^{phase}$ and the nCRF consists of eight V1-like filter outputs with four orientations and two phases $N=\left(n_{1,\theta }^{phase},n_{2,\theta }^{phase},\ldots ,n_{8,\theta }^{phase}\right),\;\theta \in \left(0^{o},45^{o},90^{o},135^{o}\right)$ and *phase* ∈ (*even, odd*) (Cagli et al., 2009; Coen-Cagli et al., 2012). Figure 1A implying statistically higher-order dependence can be well captured by a Gaussian Scale Mixture (GSM) model (Schwartz and Simoncelli, 2001; Wainwright et al., 2002; Guerrero-Colón et al., 2008; Coen-Cagli et al., 2012).

However, natural image patches are also spatially heterogeneous such as extreme instances shown in Figure 1B covering two different regions, where the dependency is quite small or even non-existent (Parra et al., 2000; Coen-Cagli et al., 2012). GSM can still describe these situations in Figure 1B by assuming independence between the outputs of CRF and nCRF. The response of MGSM can be summarized as follows (Coen-Cagli et al., 2012).


(1) $$\begin{array}{r} \begin{array}{c} \bar{R}=\bar{R_{*}}\rho \left(\xi_{*}|C,N\right)+\underset{\theta }{\sum }\bar{R_{\theta }}\rho \left(\xi_{\theta }|C,N\right), \end{array} \end{array}$$

where $\bar{R}$ indicates the estimated firing of V1 neuron, which is the summation of estimated mean response of the non-shared component $\bar{R_{*}}$ and four co-shared components $\bar{R_{\theta }}$ weighted by their corresponding posterior probabilities ρ(ξ_*_ ∣ *C, N*) and ρ(ξ_θ_ ∣ *C, N*), respectively.

According to the Bayes rule, the posterior probability can be obtained by ρ(ξ_*_ ∣ *C, N*) = ρ(ξ_*_)ρ(*C, N* ∣ ξ_*_) and ρ(ξ_θ_ ∣ *C, N*) = ρ(ξ_θ_)ρ(*C, N* ∣ ξ_θ_), where ρ(ξ_*_) and ρ(ξ_θ_), respectively, indicate the prior of the non-shared component and four co-shared components, which needs to be learned from a dataset. ρ(*C, N* ∣ ξ_*_) and ρ(*C, N* ∣ ξ_θ_) represents the likelihood of the non-shared component and four co-shared components, respectively. The specific analytic form of the likelihood is available in Coen-Cagli et al. (2012) for details.

**Figure 2:**
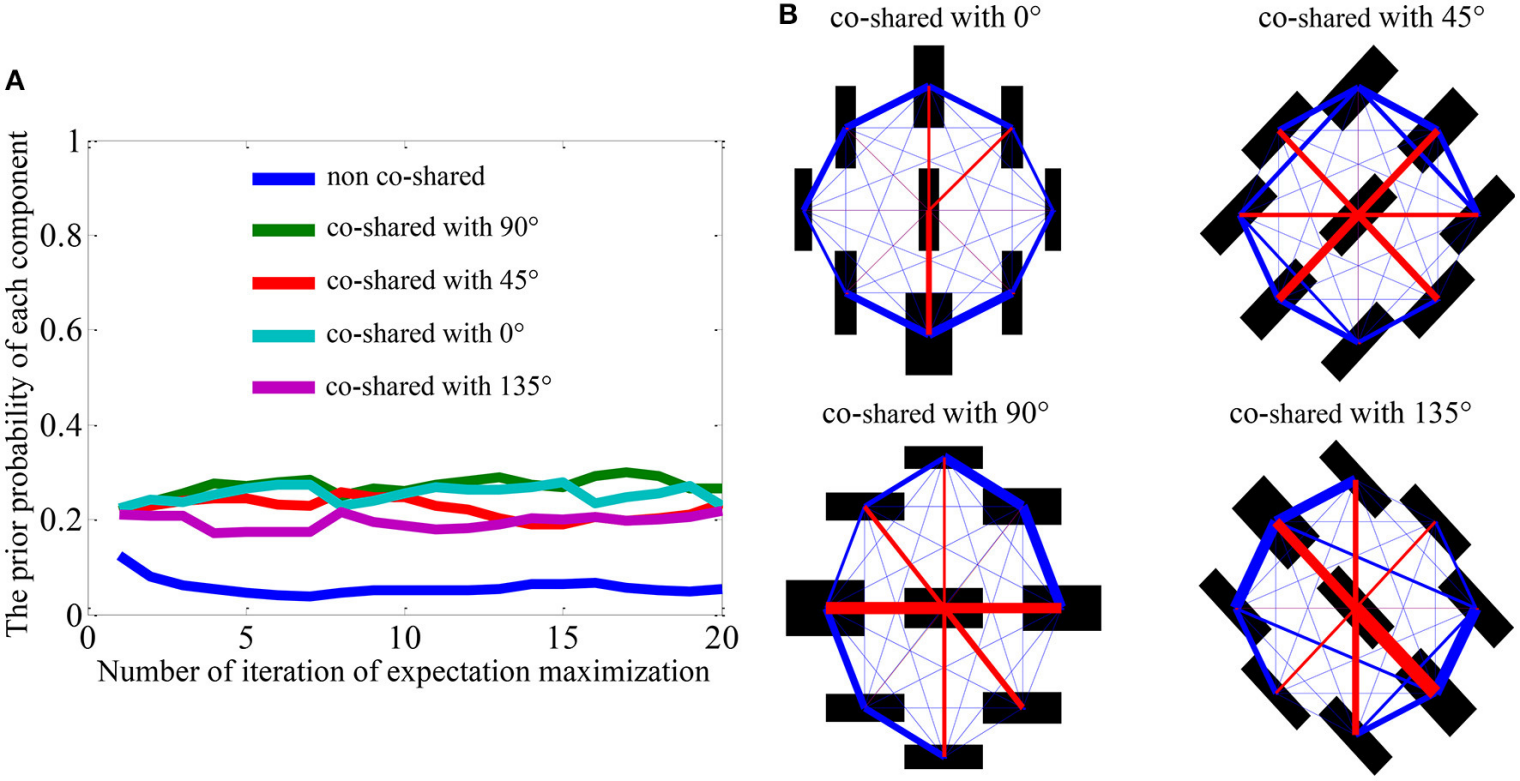
Visualization of the prior probability and covariance matrices learned from natural scenes (Coen-Cagli et al., 2016). **(A)** The prior probability of each component during the training iteration process using expectation maximization algorithm. **(B)** The covariance matrices between the CRF and nCRF outputs for four co-shared components. Black bar indicates the position of the V1-like filters in CRF and nCRF (Coen-Cagli et al., 2012). The red lines show the connecting strength between the CRF and nCRF. The blue lines show the connecting strength among nCRFs. Adapted from Coen-Cagli et al. (2012).

Essentially, MGSM in Equation (1) explains the spatial dependency and independency as seen in Figure 1 between the CRF (or center) and the nCRF (or surround), utilizing a mixture of 1) the normal GSM for cases upon which the CRF and nCRF are dependent (e.g., Figure 1A) and 2) an individual GSM model, in which the CRF and nCRF are independent (e.g., Figure 1B). For 1), *C* and *N* co-share a random variable. For example, *C* and *N* are generated through multiplying a Gaussian variable with a random variable, which is also called the mixer. As in Cagli et al. (2009); Coen-Cagli et al. (2012), we directly use four center-surround RF co-shared components $\bar{R_{\theta }}$, with θ ∈ (0^*o*^, 45^*o*^, 90^*o*^, 135^*o*^). For 2), *C* and *N* do not share a mixer (e.g., the non-shared component $\bar{R_{*}}$). The estimated mean response of the non-shared component $\bar{R_{*}}$ and the co-shared components $\bar{R_{\theta }}$ are generally given as Coen-Cagli et al. (2012) and Snow et al. (2016):


(2) $$\begin{array}{r} \begin{array}{c} \bar{R_{*}}\approx \frac{c_{\theta }}{\sqrt{{\left(c_{\theta }\right)}^{T}{\left(\Sigma_{C}\right)}^{-1}\left(c_{\theta }\right)}}, \end{array} \end{array}$$

(3) $$\begin{array}{r} \begin{array}{c} \bar{R_{\theta }}\approx \frac{c_{\theta }}{\sqrt{{\left(c_{\theta },n_{1,\theta },n_{2,\theta },\ldots ,n_{8,\theta }\right)}^{T}{\left(\Sigma_{CN}^{\theta }\right)}^{-1}\left(c_{\theta },n_{1,\theta },n_{2,\theta },\ldots ,n_{8,\theta }\right)}}, \end{array} \end{array}$$

The parameters controlling the interactions of CRF and nCRF in the MGSM model includes the covariance matrices $\Sigma_{C},\Sigma_{CN}^{0},\Sigma_{CN}^{45},\Sigma_{CN}^{90},\Sigma_{CN}^{135}$ and the prior probability ρ(ξ_*_) and ρ(ξ_θ_), θ ∈ (0^*o*^, 45^*o*^, 90^*o*^, 135^*o*^) for each component.

**Figure 3:**
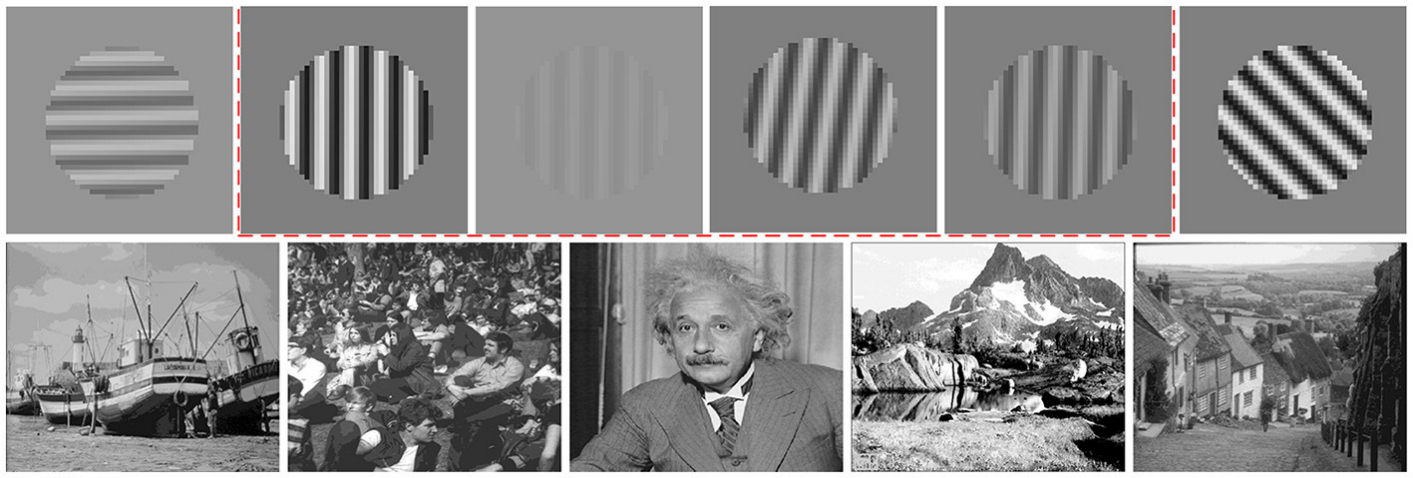
The mixed dataset containing both natural images (Coen-Cagli et al., 2016) and sinusoidal gratings under various orientations, contrasts, and phases is used to update the model using the EM algorithm, which conceptually imitates the process of the real neurophysiological experiment in which adaptation adjusts the state of the model. In the mixed dataset, the sinusoidal grating around the specific orientation (gratings with the orientation of 90^*o*^ are labeled with a red box in this example) has a higher proportion than other orientations (e.g., 0^*o*^, 45^*o*^, and 135^*o*^).

The parameters (covariance and priors) visualized in Figure 2 are obtained by training the model on 25,000 randomly sampled patches from five natural images (e.g., the second row in Figure 3). We can observe that the prior probability (Figure 2A) and covariance (Figure 2B) learned from the natural image are almost equal for each co-shared component. The prior probability of non-shared component shown in blue line is also lower than that of co-shared components shown in other color lines (Figure 2A). A possible explanation for this is that the orientation features (e.g., 0^*o*^, 45^*o*^, 90^*o*^, 135^*o*^) in a natural scene are distributed with similar probability.

## 3. Model Extensions for Orientation Adaptation in V1

The equations of extended model is firstly described. Then, we introduce how we update the parameters of the extended model using a mixed dataset. Finally, we show two strategies to fully capture the OTC adaptation effects in V1.

### 3.1. Equations for Extended Model

The extended model is summarized as


$$\begin{array}{l} \bar{R}=[WTA(\rho (\xi_{*}|C,N),\rho (\xi_{\theta }|C,N))]{\left[\bar{R_{*}},\bar{R_{\theta }}\right]}^{T},\\ \;\theta \in \{0^{o},45^{o},90^{o},135^{o}\} \end{array}$$

where [*WTA*(ρ(ξ_*_ ∣ *C, N*), ρ(ξ_θ_ ∣ *C, N*))] indicates a vector with size of 1 × 5, in which only one element equals to 1 and the rest is 0 according to WTA mechanism. ${\left[\bar{R_{*}},\bar{R_{\theta }}\right]}^{T}$ indicates a matrix with size of 5, where *M* represents the dimension of abscissa of OTC. Equation (4) indicates that only the component (e.g., ${\left[\bar{R_{*}},\bar{R_{\theta }}\right]}^{T}$) from the winning posterior probability (e.g., [*WTA*(ρ(ξ_*_ ∣ *C, N*), ρ(ξ_θ_ ∣ *C, N*))]) is accepted. In the following, we describe the approaches used to update the parameters in Equation (4) for orientation adaptation.

In specific neurophysiological tests, visual stimuli (for example, utilizing sinusoidal grating stimulation of various orientations) are used to first evaluate the OTC of a recorded V1 neuron, and the calculated OTC is viewed as a neural response before visual adaptation (i.e., before-adaptation) or without the effects of visual adaptation (Wissig and Kohn, 2012; Patterson et al., 2013; Solomon and Kohn, 2014). It is noteworthy that the neural response under this condition is equal to the normal neural response under the natural scenes, since we assume that the evolution of visual system is optimized to the natural image statistics. In order to test the visual adaptation effects on neural response, a visual stimulus with a certain orientation (e.g., a sinusoidal grating stimulation with 45^*o*^) is used to repeatedly provoke the same recorded V1 neuron within a certain time frame (so-called adaptation because the same stimulus appears to continuously stimulate the visual system). Then, the OTC of the same recorded V1 neuron after adaptation is also recorded, and we call the measured OTC after the repeatedly presented visual stimuli the neural response after adaptation (i.e., after-adaptation) (Kohn, 2007; Solomon and Kohn, 2014).

### 3.2. Updating the State Using a Mixed Dataset

In order to extend the original spatial model to further explain the temporal OTC effects, we need to modify the model in the same way as the real neurophysiological experiments. Specifically, the parameters (see Figure 2) learned from the natural images constitute a “state” of the model, and we assume this state to be the “normal state” of the model. Correspondingly, we use the same visual stimuli as used in the real neurophysiological experiments to test the OTC of the model. Then, the OTC of the model corresponds to the normal neural responses before visual adaptation (Equation 1).

We repeatedly present a sinusoidal grating with a specific orientation (e.g., 90^*o*^), as in the real neurophysiological experiments, to the model. We assume that adaptation adjusts the internal state (i.e., parameters ρ(ξ) and Σ_*CN*_) of the model indicated by Equation (4). First, we set out to determine whether adaptation may modify the “state” of the model. Second, we determine whether the modification of the “state” of the model may explain all of the OTC adaptation effects as observed in the real neurophysiological experiments. Third, we determine how exactly the modifications of the “state” of the model may predict the mechanisms behind the OTC adaptation effects.

One issue to be addressed is that we do not know which learning algorithm is used by the visual system to adjust the “state” of the neural network during adaptation with a timescale ranging from a few seconds to tens of seconds (Patterson et al., 2013). In order to address this shortcoming, we build a mixed dataset (Wainwright et al., 2002) that contains both the natural images and the gratings with various orientations as used in the neurophysiological experiments. We hypothesize that training the model on the mixed dataset using the EM algorithm can imitate the process through which adaptation modifies the “state” of the model. Specifically, we randomly generate sinusoidal grating images with various orientations, contrasts, and phases according to a two-dimensional joint distribution probability, in which the orientation meets a two-dimensional Gaussian probability distribution with (for example) a mean and variance of 90^*o*^ and 5^*o*^, respectively.

Then, the sinusoidal grating images generated according to the two-dimensional joint distribution probability are added into the natural images so as to constitute the mixed dataset, containing an ensemble of natural images and an ensemble of grating images. During the adaptation of the model, we randomly sample the image patches (26,000 image patches were used in this work) from this mixed dataset and use the sampled image patches to retrain the model from scratch. Notably, among the generated sinusoidal grating images, the grating with orientation of 90^*o*^ has higher proportion than other orientations as shown in Figure 3. We assume that this operation can mimic the visual adaptation process in the real neurophysiological experiments, where the high proportional sinusoidal gratings with certain orientation are presented to neuron during a certain timescale with higher probability than others (Benucci et al., 2013).

### 3.3. Strategies to Imitate the Process of Modeling the OTC Adaptation Effects

**Figure 4:**
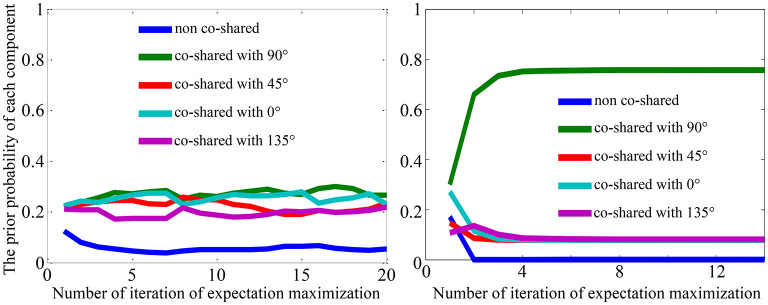
Visualization of the prior probability learned from the natural scenes (left) and learned from the mixed dataset (right) with the higher proportion of grating with the orientation of 90^*o*^ as shown in Figure 3.

Figure 4 shows the modification of the “state” of the model (i.e., parameters ρ(ξ)) after being trained on the mixed dataset. We observed that the prior probability of a co-shared component with 90^*o*^ indicated with green line (e.g., the learned prior ρ(ξ_90_) shown in the right figure of Figure 4) has been significantly increased after training the model on the mixed dataset compared to the one that has been trained on the original natural scenes, where each co-shared component has a similar prior probability (see the left figure in Figure 4). This result reflects that training the model on a mixed dataset can effectively modify the parameters of the model and thus may imitate the visual adaptation effects of neural responses.

Moreover, from Figure 4 we can infer that one of the functional effects of adaptation is to modify the prior or expectation of neural system so that the neural system can promptly follow the statistical change of the outside environment. In short, in this way, we may imitate the visual adaptation effects at various orientations on neural responses. For example, we can generate another mixed dataset that contains the grating images with higher probability at 0^*o*^, 45^*o*^, or 135^*o*^ orientations, and then train the model on these different mixed datasets to update the prior of each component.

**Figure 5:**
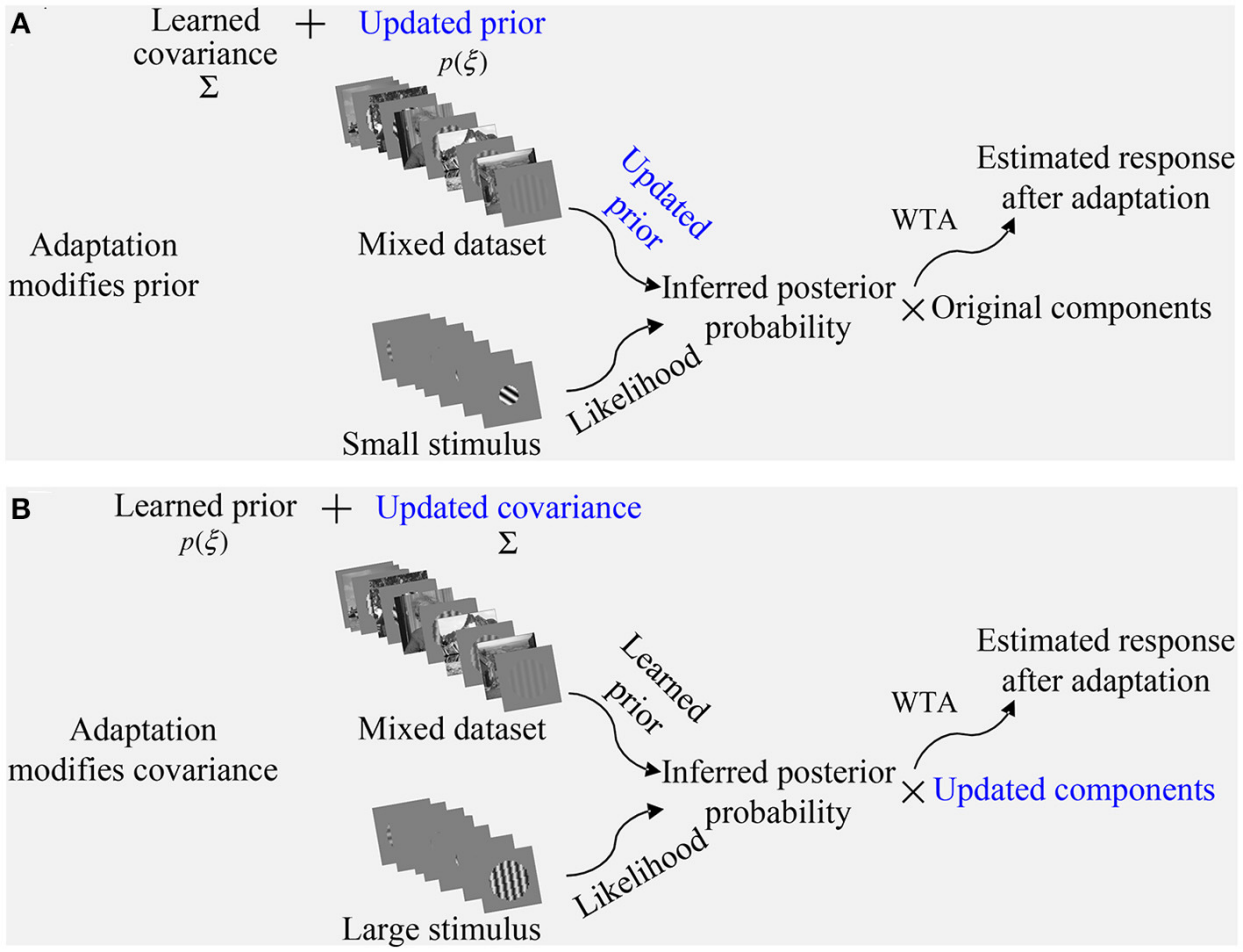
Two proposed strategies to imitate the process of modeling the visual adaptation effects on V1's orientation responses. **(A)** Adaptation modifies prior in the model. **(B)** Adaptation modifies covariance in the model, which directly results in the updated components. Two strategies of imitating the adaptation may be achieved by training the model on a mixed dataset containing both the natural images and the gratings with various orientations.

In addition to imitating the visual adaptation effects on neural responses through updating the prior in our framework, we can also update the covariance in the model as shown in Figure 2B. We will show in the following that flank adaptation of the OTC using the large grating stimuli covering both CRF and nCRF. That is, the novel attraction effects (Wissig and Kohn, 2012; Patterson et al., 2013) can not be captured by modifying the prior but can only be predicted by updating the covariance matrix in our framework. In summary, Figure 5 illustrates two strategies in our framework to imitate the process of modeling the visual adaptation effects on V1's orientation responses.

## 4. Experiments and Data

Below, we provide further information related to three key features of the working mechanisms of the extended model. Then, we compare the model simulations to experimental data on V1's OTC adaptation effects.

### 4.1. The Working Mechanisms for Orientation Adaptation

**Figure 6:**
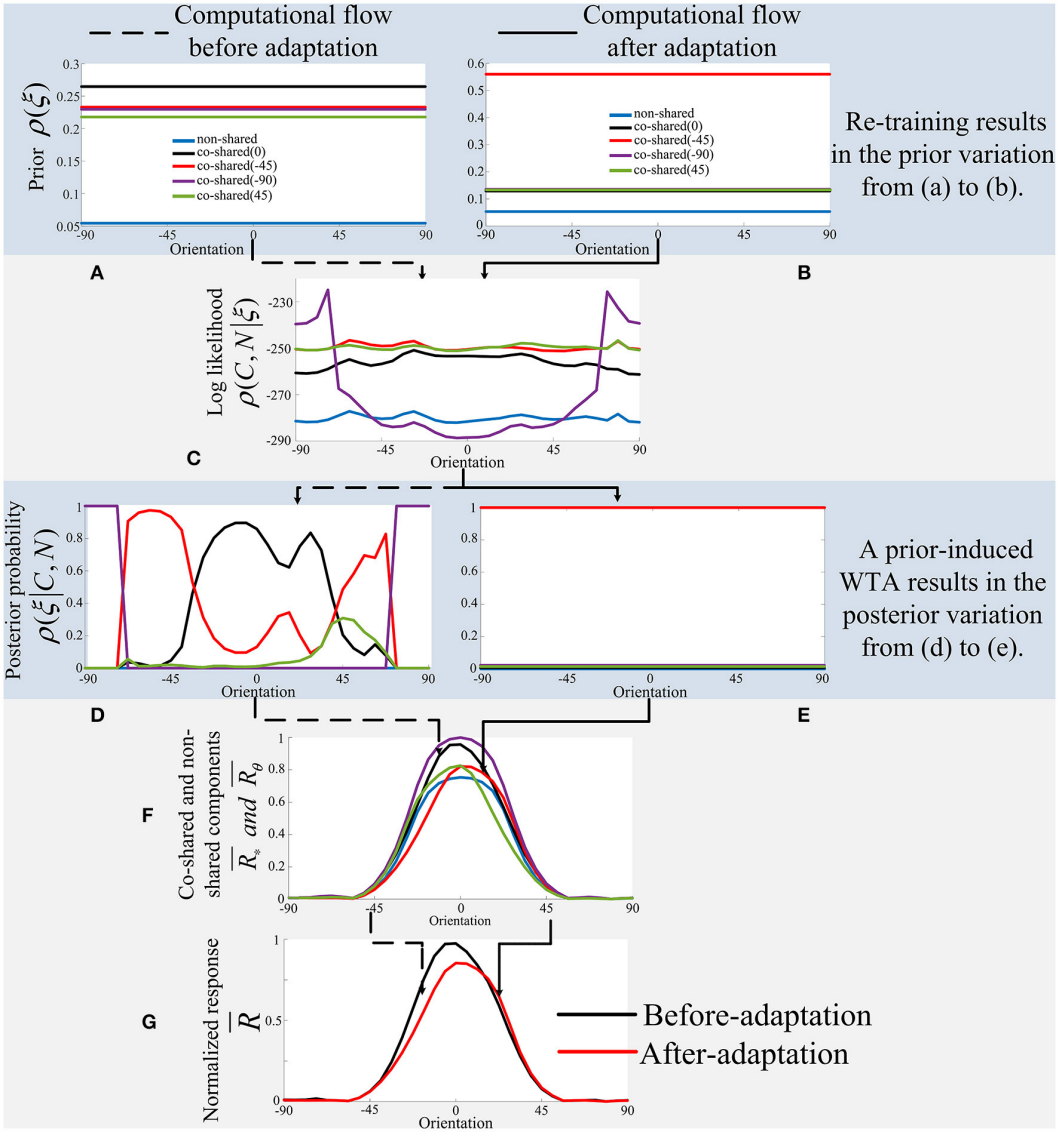
The working mechanisms of the extended model based on a prior-based WTA mechanism. In this paper, the model's and neuron's optimal orientation (i.e., 90^*o*^) is always aligned to zero for OTC visualization (e.g., the horizontal axis in each sub-panel) if there is no special statement. **(A,B)** The prior of each component before and after-adaptation. **(C)** The log likelihood of each component. **(D,E)** The posterior of each component before and after adaptation. **(F)** The normalized response of each component (e.g., “state”). **(G)** The estimated firing of V1 neuron before and after adaptation. Please see Equations (1) and (4) for mathematical computation and the main text for explanation.

Figure 6 shows the working mechanisms of the extended model, where the modification of a prior (i.e., parameters ρ(ξ)) of the model (Figure 5A) can be used to explain the orientation adaptation results observed in V1 using the small grating stimulus that just covers the CRF of a V1 neuron. The working mechanisms explain the orientation adaptation results observed in V1 using the large grating stimulus covering both CRF and nCRF, similar to Figure 6. The only difference for using the large grating stimulus is to further modify the covariance (i.e., parameters Σ_*CN*_) so as to further update the normalized response of each component (i.e., $\bar{R_{\theta }}$), as shown in Figure 5B.

In short, the working mechanisms of the model are dependent on both the prior ρ(ξ) and the likelihood ρ(*C, N* ∣ ξ) of the model, which are combined together to produce the posterior probability ρ(ξ ∣ *C, N*) = ρ(ξ)ρ(*C, N* ∣ ξ). The posterior probability further multiplies each component (e.g., $\bar{R_{*}}$ or $\bar{R_{\theta }}$) in the model, which finally produces the response of the model. For example, the estimate of firing rates $\bar{R}$ in V1 as shown in Equation (4). In Figure 6, the adaptation on the co-shared component of −45^*o*^ using a small grating is taken as an example.

#### 4.1.1. The Computational Flow Before Adaptation

For the before-adaptation information processing flow (i.e., the dashed line), the prior probabilities learned from the natural images (Figure 6A) are first combined with the likelihood (Figure 6C) to get the posterior probabilities for the co-shared component and the non-shared component (Figure 6D). It is noteworthy that the prior probability learned from the natural images (i.e., Figure 6A) for each component is quite similar. In other words, there is no one component that possesses the prior probability more than others. Thus, the posterior probability based on the combination of prior probability and likelihood for each component is also quite similar. For example, there is no one component that possesses the dominant posterior probability more than others as shown in Figure 6D. Then, the posterior probabilities are further multiplied with the normalized responses of each component (Figure 6F), and the final before-adaptation response of the model is obtained (the black line in Figure 6G).

#### 4.1.2. The Computational Flow After Adaptation

**Figure 7:**
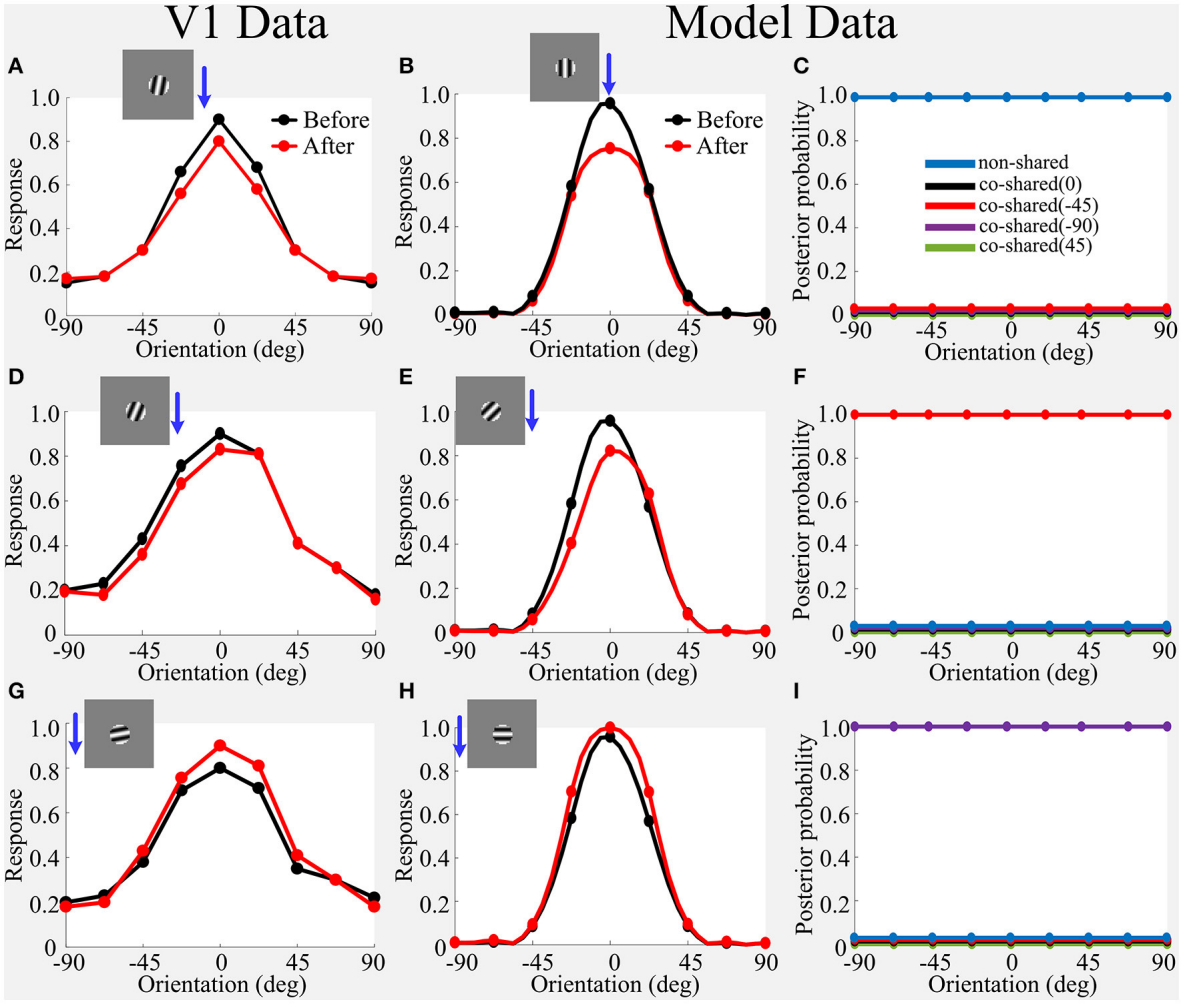
Simulated effects of adaptation on OTC in V1 using the small grating stimulus that only covers CRF. **(A,D,G)** Average OTC responses in V1 data for before-adaptation (black line) and after-adaptation (red line) when the neuron adapts at 0^*o*^−15^*o*^ away from its optimal orientation, 30^*o*^−45^*o*^ away from its optimal orientation, and 75^*o*^−90^*o*^ away from its optimal orientation, respectively. **(B)** Model prediction for before-adaptation and after-adaptation when being adapted at its optimal orientation. **(C)** The updated inferred posterior probability after model adaptation in **(B)**, where the posterior probability of non-shared component is significantly dominant. **(E,F)** Same as **(B,C)** but for model adapted at 45^*o*^ away from its optimal orientation. The updated posterior probability of co-shared component of −45^*o*^ is significantly dominant. **(H,I)** Same as **(B,C)** but for model adapted at 90^*o*^ away from its optimal orientation. The updated posterior probability of co-shared component of −90^*o*^ is significantly dominant. The blue arrowhead combining with the small sinusoidal grating roughly indicates the adapting stimulus condition for each sub-panel. The figure of V1 data is adapted from Wissig and Kohn (2012). The location of blue arrowhead is drawn slightly different from Wissig and Kohn (2012) because reported data deviates from the preferred orientation within a certain range (e.g., 0^*o*^−15^*o*^).

However, for the after-adaptation information processing flow (i.e., the solid line in Figure 6), the only difference is that the prior probability learned from the mixed dataset for the co-shared component of −45^*o*^ is significantly stronger than others (i.e., the red line in Figure 6B). This occurs since the prior for the co-shared component of −45^*o*^ is updated by modifying the model on the mixed dataset, in which the grating with orientation of −45^*o*^ occurs in a higher proportion than other orientations. Thus, the posterior probability (i.e., Figure 6E) based on the combination of prior probability (i.e., Figure 6B) and likelihood (i.e., Figure 6C) for the co-shared component of −45^*o*^ (i.e., the red line in Figure 6E) will be dominant after the updating of the prior. Then, the posterior probabilities are further multiplied with the normalized responses of each component (i.e., Figure 6F), and the final after-adaptation response of the model is obtained (i.e., the red line in Figure 6G). The main mechanism for this step is that the dominant posterior probability (i.e., the red line in Figure 6E) will select out the normalized response of co-shared component of −45^*o*^ (i.e., the red line in Figure 6F). The final results of before and after adaptation (i.e., modifying the prior of model in Equation 4) are thus obtained and are quite similar to the OTC adaptation effects as observed in V1 (see Figure 7).

#### 4.1.3. Working Mechanisms Comparison Between Snow's Model and Our Model

It should be noted that the working mechanisms of the extended model in this paper are quite different from those presented in Snow et al. (2016). First, Snow et al. updated the prior parameters for long-term adaptation simulation iteratively using the inferred posterior probability of new grating stimuli. However, the extended model in this paper updates the prior on a mixed dataset containing both natural images and grating images. Second, the inferred posterior probability in their paper is continuous (the value of inferred posterior probability is between 0 and 1) and is essentially taken as a measure of similarity between past and present stimuli, and the extent of the suppression effect relies on the inferred posterior probability. Third, their explanation for both suppression and repulsion was essentially based on the suppression mechanism relying on a flexible divisive normalization, wherein stronger similarity induces the larger inferred posterior probability and hence the stronger suppression.

In contrast, the working mechanism of our extended model (i.e., Equation 4) is based on a WTA-based state switching strategy. Before adaptation (i.e., the computational flow indicated by the dashed line in Figure 6), the components of each orientation compete with each other, and each component contributes slightly to the final orientation response. However, after adaptation (i.e., the computational flow indicated by the solid line in Figure 6), due to the significant increase of the prior of the specific orientation, a component of specific orientation finally succeeds in the competition, thus occupying a dominant position (and thus is similar to a WTA mechanism). The key point is that the modification of prior results in the variation of the posterior probability changing from Figures 6D,E). Concretely, in our model, a modification of prior for a component of specific orientation (i.e., the variation of red line from Figures 6A,B) can lead to the absolute dominance of the inferred posterior probability of the corresponding orientation (i.e., the variation of red line from Figures 6D,E). Hence, adaptation plays a functional role in a WTA mechanism to select out the normalized neural response component with the dominant posterior probability regardless of the similarity between the adaptor and the test stimulus (e.g., the OTC response labeled with the red line in Figure 6G is obtained by G = E^*^F according to Equation 4). In short, the inferred posterior probability is no longer treated as a measurement with continuous value between [0, 1] to determine the size of suppression effect, but acts as a WTA mechanism through taking a discrete value of either 0 or 1 (Equation 4). For example, the inferred posterior probability of the corresponding orientation (i.e., the red line in Figure 6E) is always equal to 1 across all orientations. In contrast, the inferred posterior probability of other orientations (i.e., the other color lines in Figure 6E) are always equal to 0 across all orientations.

Furthermore, Snow's model does not include nCRF and hence cannot explain the facilitation and attractive shift effects of OTC after adaptation. However, the extended model can primarily capture the disinhibition effects due to inclusion of nCRF (Coen-Cagli et al., 2012) and hence can explain the facilitation and attractive shift effects. The result of our model framework is that there are several states (e.g., the non-shared and co-shared components in Figure 6) in the framework, and an a priori change leads to switching among different states. This explanation seems to be more reasonable than measuring the inhibition based on the similarity of the past stimulus and the current stimulus. We found that a small change of priors will suddenly lead to the value of posterior probabilities to be either 0 or 1 (e.g., Figure 6E), and thus our framework is not able to produce the posterior with continuous value as obtained by Snow et al. (2016).

It should be noted that each component of the model (e.g., Figure 6F) is also sensitive to the modification of the covariance matrix of the model learned from the mixed dataset (see **Figure 10** for more details). In the next section, we will show that how the updating of prior and covariance of the model can effectively capture all of the primary OTC adaptation results in V1 (Wissig and Kohn, 2012; Patterson et al., 2013).

### 4.2. Adaptation Modifying State Explains the OTC Adaption Effects on V1 Neuron

Our framework predicts that OTC adaptation leads to the state switch of cortical network. The modification of state can be achieved by exposing the model to a mixed dataset, where a grating with specific orientation (for example, when the orientation is similar to the adapter) has the higher proportion than other orientations.

#### 4.2.1. Prior-Based WTA Predicts OTC Adaptation Covering CRF

In the physiological experiments (Wissig and Kohn, 2012; Patterson et al., 2013), the adaptation using the small grating that just covers CRF of a V1 neuron induces the typical suppressive, repulsive, and orthogonal enhancement effects in the OTC of V1 neuron (Figures 7A,D,G). Here, we show that the spatial model based on prior-based WTA can qualitatively capture these results.

**(1) Suppressive Effect**. In our framework, OTC of the model without the modification of a prior is used as the baseline (i.e., before-adaptation). To measure the effects of adaptation on the model's OTC, the tested OTC of the model with the modification of a prior is used. Our framework clearly reproduces this suppressive effect (Figure 7B), which can be explained by the working mechanisms of prior-based WTA in that the dominant inferred posterior probability of the specific component is induced by the modification of the prior (see the working mechanisms in Figure 6). The posterior probability inferred by the model for the non-shared component (Figure 7C) is significantly increased with the modified prior of the co-shared component with 0^*o*^ (i.e., adapted to the model's optimal orientation; the model's optimal orientation (90^*o*^) is aligned to 0^*o*^ for visualization), which results in the WTA-based selection of the normalized response for the non-shared component. Interestingly, the increase of the prior of the co-shared component with 0^*o*^ results in the final selection of the non-shared component. Intuitively, we might expect that increasing the prior of the co-shared component with 0^*o*^ can lead to the increase in the inferred posterior probability of dependence of the co-shared component with 0^*o*^ when using the small grating (the black line in Figure 7C).

However, the truth is that increasing the prior of the component co-shared with 0^*o*^ cannot increase the inferred posterior probability of the dependence, but would rather inversely reduce the dependence between CRF and nCRF, and would result in the dominance of the inferred posterior probability of the non-shared component (the blue line in Figure 7C). This means that the suppression effect after adaptation is not from the component co-shared with 0^*o*^ that produces the suppression dependent on the surround nCRF signals according to Equation (3), but from the pure CRF-based response and suppression [i.e., the response of the non-shared component does not contain any nCRF-dependent suppression according to Equation (2)]. This result predicts that adaptation and testing using a small grating can transform a model with a co-shared component state to a model with a non-shared component state, and hence constitute a disinhibition effect in which the non-shared component does not contain any nCRF signals.

Our explanations for the typical suppression effect using the small grating are different from Snow et al. (2016), wherein the authors explained the suppression effect based on the inferred posterior probability of dependence between the past and the present stimuli, which in turn determined the strength of suppression induced by the divisive normalization signals recruited by the past stimuli to the response of present stimuli. The suppression effect of Snow et al. (2016) is essentially a consequence of the orientation-specific suppression mechanism (i.e., strongest suppression when the orientation of adapting stimulus is similar to the orientation of test stimulus). In contrast, the suppression effect of our framework is from the switch between two model states (e.g., from a co-shared component state to a non-shared component state).

Furthermore, the inferred posterior probability before adaptation in our framework (the black and red lines in Figure 6D) is very similar to the inferred posterior probability after adaptation of Snow et al. (2016) (e.g., Figures 4C,F). Their results are based on the inferred posterior probability where the suppression is the strongest at 90° or 45°, and the suppression is the weakest on the two sides of 90° or 45°. However, based on the CRF-nCRF model, our framework produces the suppression result because the model selects a non-shared component state, which seems to mean either that adaptation leads to the collective silence of peripheral nCRF neurons or that there is no CRF-nCRF correlation.

**(2) Repulsion Effect**. We further investigated the case when the adapter is adapted at 45^*o*^ away from the neuron's preferred orientation (Figure 7D; Wissig and Kohn, 2012). The repulsion effect can also be captured qualitatively by our framework (Figure 7E), because the dominant posterior probability was determined by the high prior of the co-shared component of −45^*o*^, which leads to the switch between two states (e.g., from a co-shared component of 0^*o*^ to a co-shared component of −45^*o*^) and the final selection of the normalized response of −45^*o*^ in the model. Our explanations for the repulsion effect again are different from the orientation-specific suppression mechanism proposed in Snow et al. (2016). The slight difference between V1 data and model prediction at the adapted orientation (−30^*o*^ in Figure 7D vs. −45^*o*^ in Figure 7E) occurs because the co-shared components in the model only imitate four filters' orientations (0^*o*^, 45^*o*^, 90^*o*^, 135^*o*^), and hence the model cannot finely simulate adaptation at other orientations (e.g., 30^*o*^) (Coen-Cagli et al., 2012; Snow et al., 2016).

**(3) Facilitation Effect**. We next consider the third situation of adaptation using a small grating whose orientation is orthogonal to neuron's preferred orientation. In this case, OTC is enhanced after adaptation compared to the original OTC before adaptation, which is the so-called orthogonal enhancement (Figure 7G; Wissig and Kohn, 2012). The model developed in Snow et al. (2016) cannot capture the interesting data of orthogonal enhancement, as their model is essentially a divisive normalization-based suppression model. However, the orthogonal enhancement effect can be still captured qualitatively by our framework (Figure 7H), because the dominant posterior probability was determined by the high prior of the co-shared component of −90^*o*^, which leads to a switch between two states (e.g., from a co-shared component of 0^*o*^ to a co-shared component of −90^*o*^) and the final selection of the normalized response of −90^*o*^ in the model.

In our framework, we assume that the adapter will induce a strong prior for the specific orientation, regardless of the normalized response component (e.g., Figure 6F) and the likelihood (e.g., Figure 6C) of each component (e.g., the non-shared components and co-shared components for 0^*o*^, −45^*o*^, −90^*o*^, and 45^*o*^) in Figure 6. The posterior probability after adaptation is significantly dominant for the specific orientation due to the combination of the strong prior and the unchanged likelihood. Because the posterior probability is further used to multiply each normalized neural response component (e.g., Figure 6F), adaptation is functionally implementing a WTA mechanism to select out the normalized neural response component with the dominant posterior probability as the final neural response (e.g., Equation 4), regardless of the similarity between adapting stimulus and test stimulus (e.g., 0^*o*^ in Figure 7B, −45^*o*^ in Figure 7E and −90^*o*^ in Figure 7H). Our modeling framework thus provides a prior-induced WTA mechanism for explaining the orientation-specific adaptation using a small grating (Solomon and Kohn, 2014; Snow et al., 2016).

#### 4.2.2. Prior-Based WTA Partly Predicts OTC Adaptation Covering CRF and nCRF

**Figure 8:**
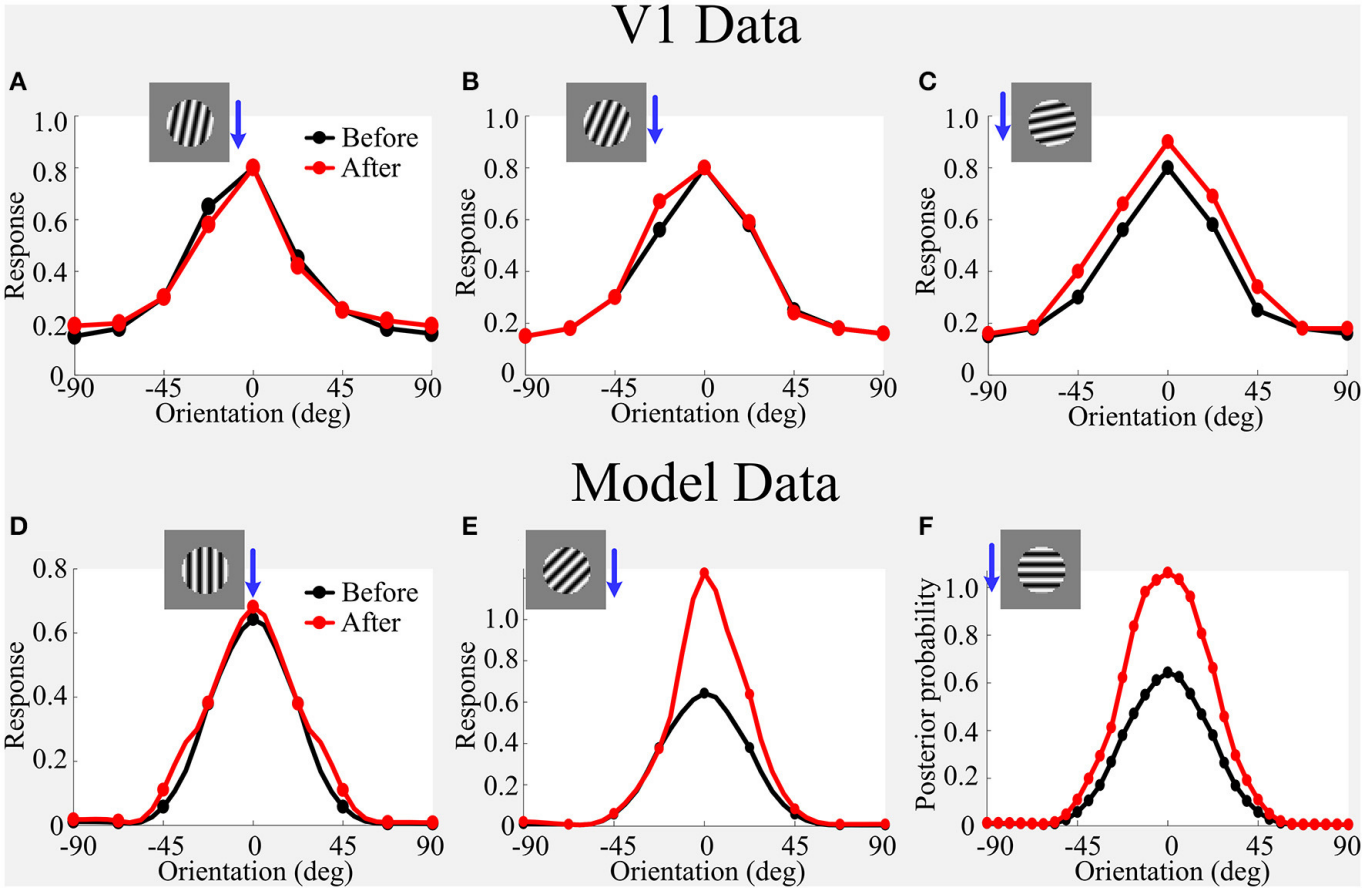
**(A–F)** Simulated effects of adaptation on OTC using the large grating stimulus that covers both CRF and nCRF. Blue arrowhead combining with the large sinusoidal grating roughly indicates the adapting stimulus condition for each sub-panel. Refer to Figure 7 for explanations of V1 Data vs. Model data. The figure of V1 data is adapted from Wissig and Kohn (2012); Patterson et al. (2013).

The prior-based WTA-induced model state switch also can explain most of the OTC adaptations under large grating stimulus covering both CRF and nCRF as shown in Figure 8. In short, responses after adaptation when the adapter orientation is matched to the preferred orientation are maintained (Figure 8A). Responses after adaptation when the adapter orientation is away from the preferred orientation attract OTC toward the adapter (Figure 8B). Finally, responses after adaptation when the adapter orientation is orthogonal to the preferred orientation are enhanced (Figure 8C). Our framework based on the modification of prior qualitatively captured both the maintained and enhanced effects (Figure 8A vs. Figure 8D and Figure 8C vs. Figure 8F).

However, our model based on the modification of prior cannot capture the attraction effect using the large grating stimulus as the adapter covering both CRF and nCRF (Figure 8B vs. Figure 8E), where our model produces the combinational effects containing both repulsion and enhancement. In order to capture the attraction effect, this framework may further require the changes in the connectivity of CRF and nCRF (Coen-Cagli et al., 2012; Snow et al., 2016).

Hence, we further assume that adaptation using the large grating stimulus covering both the CRF and nCRF is not only modifying the prior during adaptation but also possibly reflecting changes in the covariance (Figure 5B) based on updating the connectivity between CRF and nCRF during adaptation. In the following, we will show how manually modifying covariance in the model can capture the novel attraction effects on V1 when using the large grating stimulus covering both CRF and nCRF as the adapter.

#### 4.2.3. The Combined Influence of Altered Variance and Covariance Predicts Attraction Effects

**Figure 9:**
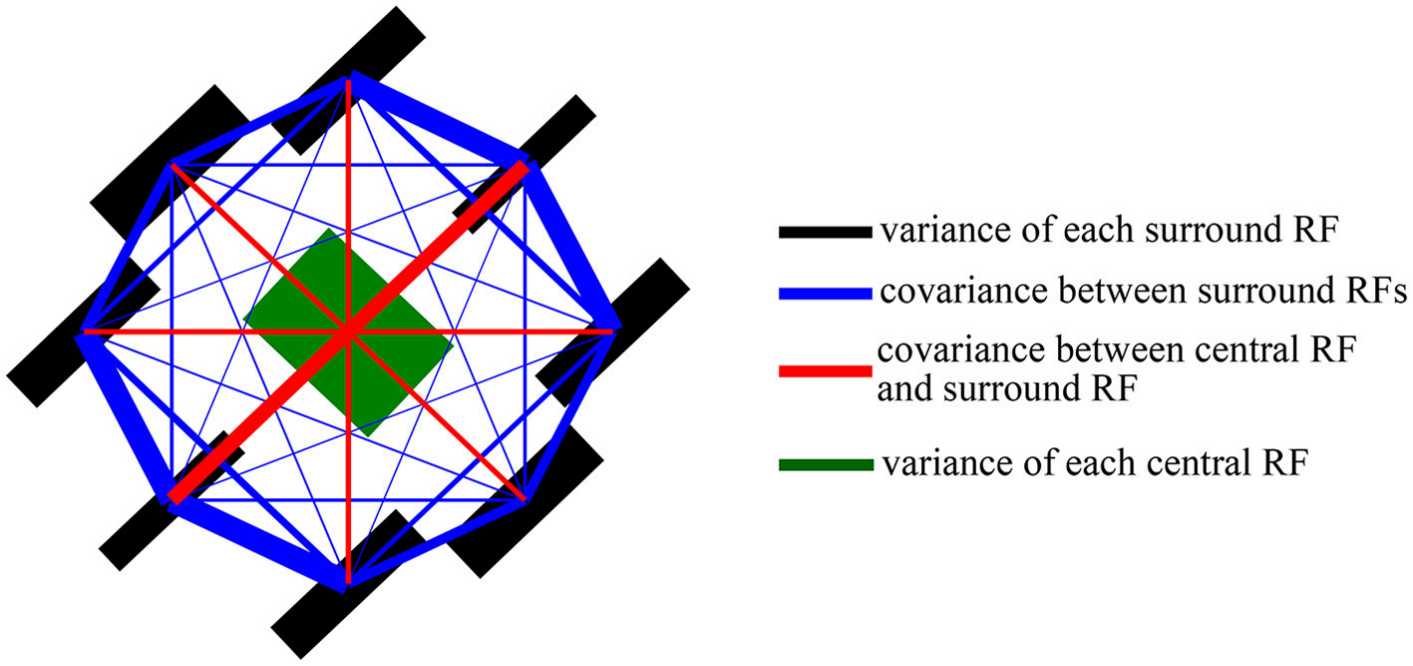
Visualization of the covariance matrix for the co-shared component of 45^*o*^ learned from scenes. The only difference compared with Figure 2 is that we further visualize the variance of the CRFs (e.g., the thickness of green lines is proportional to the variance of CRFs).

To understand how the framework produces the attraction effects by manually modifying the covariance, the learned covariance matrices (i.e., $\Sigma_{CN}^{45}$) are visualized in Figure 9. For example, the black bars in Figure 9 are the learned variances for the model of the co-shared component of 45^*o*^, which reflect the strength of normalization from nCRFs. The framework models nCRF using eight V1-like RFs. The green lines are the learned variance, reflecting the strength of normalization from CRFs (e.g., the CRF contains four V1-like RFs with different preferred orientations Coen-Cagli et al., 2012). The variance and covariance together form the weights that adjust signals from CRF and nCRF when the responses of center and surround RFs are excited, which can induce the orientation-specific enhancement and suppression during adaptation.

**Figure 10:**
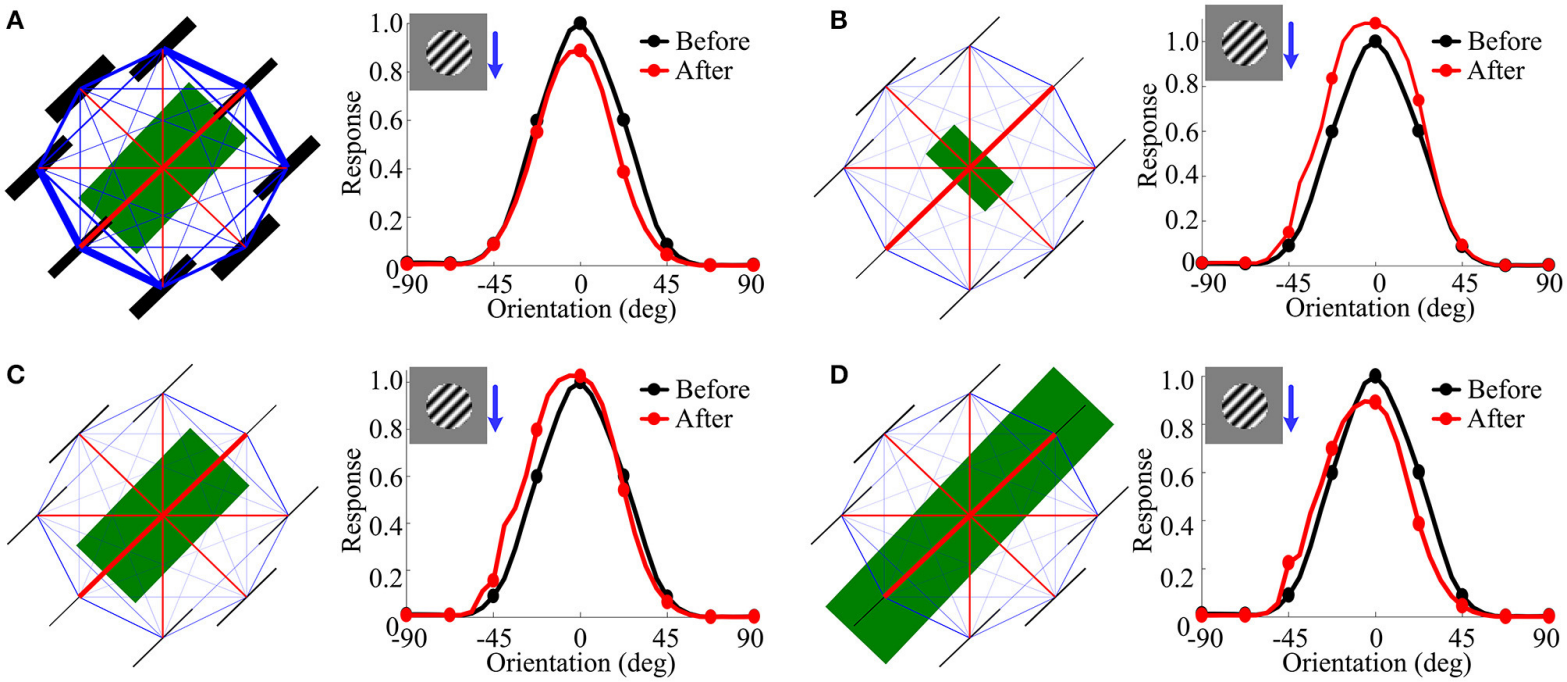
Visualization of each part of covariance matrix reproducing the attraction effects. **(A)** Increasing the variance of central RFs for orientations of 0^*o*^, 90^*o*^, and 135^*o*^ reproduces the attraction effects as observed in V1 (Patterson et al., 2013). **(B)** Weakening the variance and covariance of surround RFs reproduces the attraction effects as observed in V1 to some extent (Patterson et al., 2013). **(C)** The combined influence of altered variance and covariance as in **(A,B)** clearly reproduces the attraction effects as observed in V1. **(D)** The effects of further double-scaling the variance of central RFs for orientations of 0^*o*^, 90^*o*^, and 135^*o*^ in **(C)**. The blue arrowhead combining with the large sinusoidal grating indicates the adapting stimulus condition for each sub-panel.

**(1) Enhanced suppression within CRFs**. In all of the experiments, we use the same prior as the previous part. Figure 10 shows the function of each part of the covariance matrix during the reproduction of the attraction effects as observed in V1 when using the large grating stimulus covering both CRF and nCRF as the adapter. We observed that only increasing the variance of CRFs (i.e., comparing the bar thickness of green lines in Figures 9, 10A) for orientations of 0^*o*^, 90^*o*^, and 135^*o*^ can capture the attraction effects as observed in V1 (Figure 10A). The reason is that increasing the variance of CRFs for the orientations of 0^*o*^, 90^*o*^, and 135^*o*^ is functionally equal to enhancing the normalization signals within CRFs from the corresponding orientations (e.g., 0^*o*^, 90^*o*^, and 135^*o*^), which finally results in further suppression within CRFs. Thus, we observed the clear suppression of the right part of OTC in Figure 10A after adaptation (e.g., red line). This enhanced suppression from CRFs that leads to the attraction effects observed in the model is essentially different from the attraction effects experimentally observed in V1 (Wissig and Kohn, 2012; Patterson et al., 2013), where the attraction effects were generally explained by an adaptation induced weakening of surround suppression (i.e., weakening of nCRFs).

**(2) Weakening suppression from nCRFs**. In order to test whether our framework can capture the attraction effects by only weakening of surround suppression as observed in V1 (Wissig and Kohn, 2012; Patterson et al., 2013), we decreased the variance and covariance of surround RFs in the model so as to imitate the mechanism of weakening surround suppression as suggested by Wissig and Kohn (2012); Patterson et al. (2013). The weakening of the surround suppression works to decrease the normalization signals from the corresponding orientation (e.g., 45^*o*^). Figure 10B shows the results of weakening the surround suppression. We observed that the weakening of the surround suppression can only reproduce the attraction effects as observed in V1 to some extent but cannot fully explain the observed attraction effects in V1 as observed by Wissig and Kohn (2012) and Patterson et al. (2013). For example, the left part of OTC represented by the red line in Figure 10B is further enhanced after adaptation following the decrease of the normalization signals, which is similar to Figure 9B in Wissig and Kohn (2012). However, only weakening (or disinhibition) of the surround suppression cannot increase the normalization signals for other corresponding orientations in the CRFs (e.g., 0^*o*^, 90^*o*^, and 135^*o*^), and thus cannot enhance the suppression of the right part of OTC after adaptation as reflected in Figure 10A. The weakening of the surround suppression mechanism also partly leads to the facilitation of the right part of OTC after adaptation to some extent (see red line in Figure 10B), which was not observed in V1 adaptation experiments using the large grating stimulus (Wissig and Kohn, 2012; Patterson et al., 2013).

**(3) Combination of two mechanisms**. Figure 10C shows the results of the combined influence of altered variance (specifically, increasing the variance of CRFs) and covariance (decreasing the variance and covariance of nCRFs) as shown in Figures 10A,B together, which clearly reproduce the attraction effects as observed in V1 (Wissig and Kohn, 2012; Patterson et al., 2013). Figure 10D shows the more obvious attraction effects by doubly increasing the variance of CRFs (e.g., the bar thickness of green lines) for orientations of 0^*o*^, 90^*o*^, and 135^*o*^ in Figure 10C. This effect further stresses the importance of increasing the normalization signals within CRFs and hence enhancing the suppression directly from the CRFs when producing the attraction effects in V1 after adaptation.

The results obtained by our framework indicate that in order to predict the attraction effects, only the explanation of weakening (or disinhibition) of the surround suppression is not enough (Wissig and Kohn, 2012; Patterson et al., 2013; Solomon and Kohn, 2014). Adaptation using the large grating stimulus not only leads to the adaptation-induced weakening of surround suppression but may also result in the adaptation-induced enhancement of center suppression. In summary, for large grating stimuli based flank adaptation of the OTC in V1, the effect indicates the comprehensive impact of the enhanced center suppression from the non-adapted orientations within the CRF and the weakened surround suppression from the adapted orientation within the nCRF; the former yields the response reduction for the non-adapted orientations (see Figure 10A), and the latter results in the facilitation for the adapted orientation (see Figure 10B) after adaptation. These two factors combine to form the attractive shift effect. Furthermore, the decrease of peak response and shift switching from repulsion to attraction of OTC are mainly dependent on the strength of enhanced responses for the non-adapted orientations within CRFs (i.e., enhancement of the center suppression).

## 5. Discussion and Conclusion

We designed a framework to study the parameters of a scene statistics-dependent spatial model to explain the orientation adaptation phenomena observed in V1 (Coen-Cagli et al., 2012). We extended this model by updating the parameters based on a mixed dataset that included both scene statistics and synthetic statistics, such as grating images widely adopted in neurophysiological experiments (Wissig and Kohn, 2012; Patterson et al., 2013; Solomon and Kohn, 2014). Results show that the extended model has been able to capture all of the OTC adaptation effects observed in neurophysiological experiments.

In order to capture the physiological data, three specific predictions were necessary. First is the prediction that the OTC adaptation is sensitive to the variation of a prior. Second is the prediction that there is prior-induced WTA that selects through successful competition one component from a pile of normalized response components in the model. Third is the prediction that adaptation using a large grating stimulus covering both CRF and nCRF induces the comprehensive effects of enhanced suppression within CRFs and weakening of surround suppression from nCRFs.

The enhanced suppression within CRFs may be explained by the non-specific suppression within the CRF (Morrone et al., 1982; Bonds, 1989; DeAngelis et al., 1992; Heeger, 1992; Carandini et al., 2005), where the RFs of multiple neurons with various orientation selectivity overlap, and the responses of a neuron can be inhibited by pooling the responses of multiple neurons. The CRF in the MGSM model contains four overlapping V1-like filters with four different orientations and two different phases. Our results indicate that adaptation using the large grating induced a similar effect of enhanced nonspecific suppression, and the observed attractive effect after adaptation in V1 mainly resulted from the adaptation-induced nonspecific suppression mechanism. The possible physiological mechanisms implementing the changes in connectivity of covariance during adaptation may raise from stimulus dependent variation of lateral connectivity and strength among neurons (Nauhaus et al., 2009; Coen-Cagli et al., 2015) or fast conductance changes of neurons (Connor, 1978).

The working mechanism of our extended model maybe quite different from the previous work of Coen-Cagli et al. (2012) and Snow et al. (2016), in which the authors stressed the importance of the strength of the stimulus-dependent normalization based on measuring the feature similarity between center and surround (Coen-Cagli et al., 2012) or between past and present (Snow et al., 2016). In contrast, our extended model is based on a prior-induced WTA mechanism that drives a switch among model states. Consistent with traditional findings that neurons in V1 receive intracortical modulation (Carandini et al., 2005) and hence constitute a neural network that can respond to different stimuli attributes (e.g., different orientations), the concise MGSM model contains five different components (a non-shared component and four co-shared components with orientations of 0^*o*^, 45^*o*^, 90^*o*^, and 135^*o*^), and each one responds to a specific model state (i.e., orientation). Therefore, a prior-induced WTA mechanism that drives a switch among different model states may be more reasonable than a mechanism based on measuring the feature similarity for the following reasons.

1) The structure of the extended framework is physiologically more consistent with the basic neural substrates (in other words, neurons receiving a pool of intracortical signals produced by neurons with various orientations and spatial frequencies constitute a neural network) (Morrone et al., 1982; Bonds, 1989; DeAngelis et al., 1992).

2) More and more studies have indicated a link between adaptation and attention (Boynton, 2004; Solomon and Kohn, 2014); both are major mechanisms modulating the sensitivity of the brain to visual stimuli in temporal and spatial dimensions. Although WTA mechanisms are not clearly linked with adaptation and attention, two prior examples in the literature did find a potential role for WTA in adaptation and attention. Lee et al. discovered that attention can induce WTA competition among visual RFs (Lee et al., 1999). Jin et al. directly exploited a WTA mechanism to obtain the perceived orientation from a population coding of neurons (Jin et al., 2005), which was further used to measure whether the orientation adaptation responses of V1 may predict the tilt aftereffect measured by a psychophysics experiment.

3) Adaptation during a few seconds or tens of seconds may be predictive (Chopin and Mamassian, 2012)—that is, not calibrating the neural system to the recent history as suggested by Snow et al. (2016), wherein the author used the recent posterior as the updated prior (e.g., the prior is updated only using a recent set of grating stimulus)—but rather than estimating the prior from the remote history, which is similar to our extended MGSM model. In the MGSM model, the prior is updated on a mixed dataset containing both natural images and grating images (e.g., a kind of remote history) and then is used as the reference to select out the corresponding model state (e.g., through a prior induced WTA mechanism). In summary, the major novel contribution of this work is to establish the role of prior-induced WTA on orientation adaptation effects in V1.

Finally, Shushruth et al. (2013) has shown that nCRF can be further divided as separate entities of near vs. far surround. They have been shown to have distinct surround suppression magnitudes and mechanisms. Hence, how adaptation interacts with two separate entities was not clear from previous work (Wissig and Kohn, 2012; Patterson et al., 2013; Solomon and Kohn, 2014). The CRF-nCRF model may be further extended to explore these subtle key points for insightful exploration in the future.

## Data Availability Statement

The original contributions presented in the study are included in the article/supplementary material, further inquiries can be directed to the corresponding author. The source code of MGSM used in this work to visualize the parameters and natural image dataset are directly from Dr. Ruben Coen-Cagli, which are also available in http://dx.doi.org/10.6080/K0JM27JZ.

## Author Contributions

SBG performed the research and wrote the first draft of the manuscript. SBG and XL acquired funding for research. Both authors contributed to the article and approved the final submitted version.

## Funding

This study was partly supported by the National Natural Science Foundation (grant nos. 61806134 and 62076170), Sichuan Key Research and Development Program (grant no. 2020YFG0324), the National Key R&D Program of China (2020AAA0104500), and the Fund of Sichuan University Tomorrow Advancing Life. The funders had no role in study design, data collection and analysis, decision to publish, or preparation of the manuscript.

## Conflict of Interest

The authors declare that the research was conducted in the absence of any commercial or financial relationships that could be construed as a potential conflict of interest.

## Publisher's Note

All claims expressed in this article are solely those of the authors and do not necessarily represent those of their affiliated organizations, or those of the publisher, the editors and the reviewers. Any product that may be evaluated in this article, or claim that may be made by its manufacturer, is not guaranteed or endorsed by the publisher.
